# Spreading Patterns of the Influenza A (H1N1) Pandemic

**DOI:** 10.1371/journal.pone.0017823

**Published:** 2011-03-31

**Authors:** Sergio de Picoli Junior, Jorge Juarez Vieira Teixeira, Haroldo Valentin Ribeiro, Luis Carlos Malacarne, Ricardo Paupitz Barbosa dos Santos, Renio dos Santos Mendes

**Affiliations:** 1 Departamento de Física, Universidade Estadual de Maringá, Maringá, Paraná, Brazil; 2 Departamento de Análises Clínicas e Biomedicina, Universidade Estadual de Maringá, Maringá, Paraná, Brazil; 3 National Institute of Science and Technology for Complex Systems, Rio de Janeiro, Rio de Janeiro, Brazil; Instituto Butantan, Brazil

## Abstract

We investigate the dynamics of the 2009 influenza A (H1N1/S-OIV) pandemic by analyzing data obtained from World Health Organization containing the total number of laboratory-confirmed cases of infections - by country - in a period of 69 days, from 26 April to 3 July, 2009. Specifically, we find evidence of exponential growth in the total number of confirmed cases and linear growth in the number of countries with confirmed cases. We also find that, i) at early stages, the cumulative distribution of cases among countries exhibits linear behavior on log-log scale, being well approximated by a power law decay; ii) for larger times, the cumulative distribution presents a systematic curvature on log-log scale, indicating a gradual change to lognormal behavior. Finally, we compare these empirical findings with the predictions of a simple stochastic model. Our results could help to select more realistic models of the dynamics of influenza-type pandemics.

## Introduction

The spread of infectious diseases is a threat to the world public health and a subject of great scientific interest. Influenza viruses, for example, circulate around the world every year and seasonal influenza is one of the most worrying respiratory infections of humans [1]. From time to time new strains of influenza virus emerge and cause large-scale global pandemics – such as the 2009 influenza A (H1N1/S-OIV). The dynamics of influenza-type pandemics has been the focus of several scientific works which may provide necessary information to deal with future pandemic events [2]–[9]. However, conventional modeling techniques are usually only able to provide a general idea of how a pandemic might evolve, since crucial information concerning model parameters is generally unavailable.

## Methods

Here, we investigate the dynamics of influenza-type pandemics using techniques of statistical physics typically applied in the study of complex systems. We analyze a database - from World Health Organization - containing laboratory-confirmed cases of influenza A (H1N1/S-OIV) infections around the world [10]. Specifically, we analyze the total number of laboratory-confirmed cases - by country - in a period of 69 days, from 26 April to 3 July, 2009.

We search for patterns of spread for the virus influenza A (H1N1/S-OIV). Basically, we investigate the growth in the total number of confirmed cases, the growth in the number of countries with confirmed cases and the cumulative distribution of cases among countries. We also propose a simple stochastic model which reproduces the main empirical findings obtained from the analysis of the database on the 2009 influenza A (H1N1/S-OIV) pandemic.

## Results

**Figure 1:**
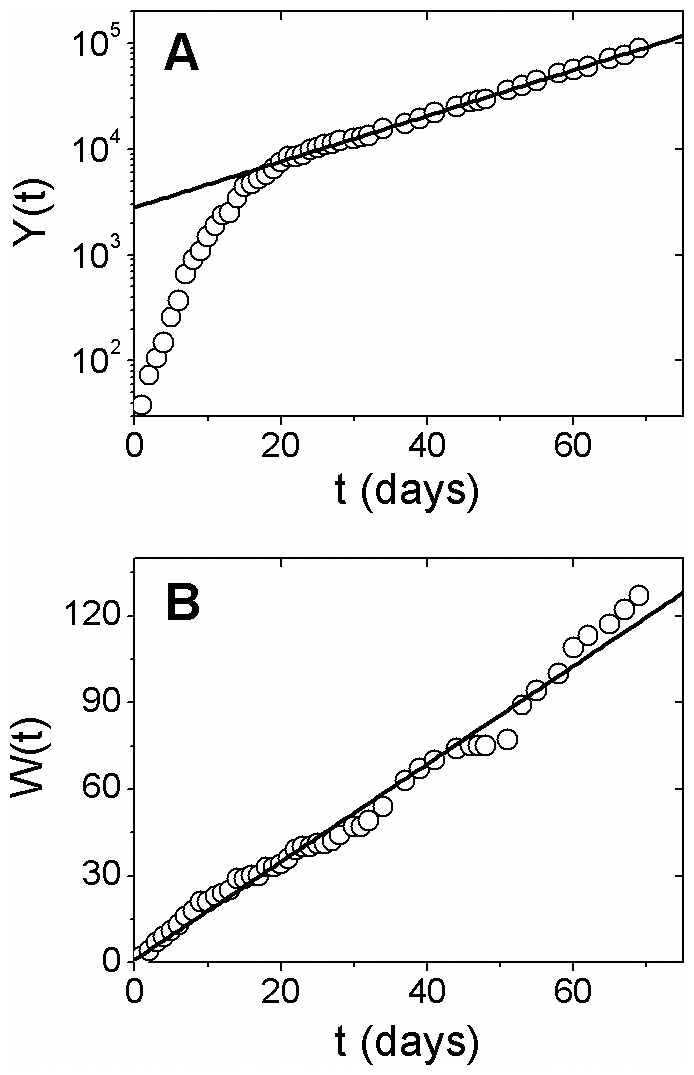
Temporal evolution of cases and countries with confirmed cases. (**A**) The world total number of influenza A (H1N1) laboratory-confirmed cases, *Y*(*t*), on mono-log scale, for the period of 69 days, from 26 April to 3 July, 2009. The solid line is given by 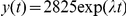, with 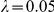. (**B**) The total number of countries with confirmed cases, *W*(*t*), for the same period. A linear least-square fit to the data (solid line) suggests 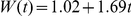.

First, we consider the time evolution of the global number of confirmed cases, *Y*(*t*). At a given time *t*, the total number of cases *Y* is given by the sum of *y* over all countries. In Fig. 1A we show *Y*(*t*) in comparison with an exponential curve, given by the equation 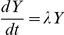, with 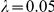 (compare with results reported in ref. [11]). This result suggests that the total number of confirmed cases exhibits an exponential growth. However, a systematic deviation of the exponential behavior can be observed for smaller times (*t* < 20 days). In addition, Fig. 1B shows the time evolution of the total number of countries with confirmed cases, *W*(*t*). Observe that *W*(*t*) exhibits approximately a linear growth in all the period considered.

Next, we investigate the distribution of confirmed cases among countries and its time evolution. In order to reduce statistical fluctuations, it is common to consider the cumulative distribution 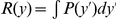. Here, *y* is the number of confirmed cases in a given country and *P*(*y*) is the probability distribution function (PDF) of *y*.

**Figure 2:**
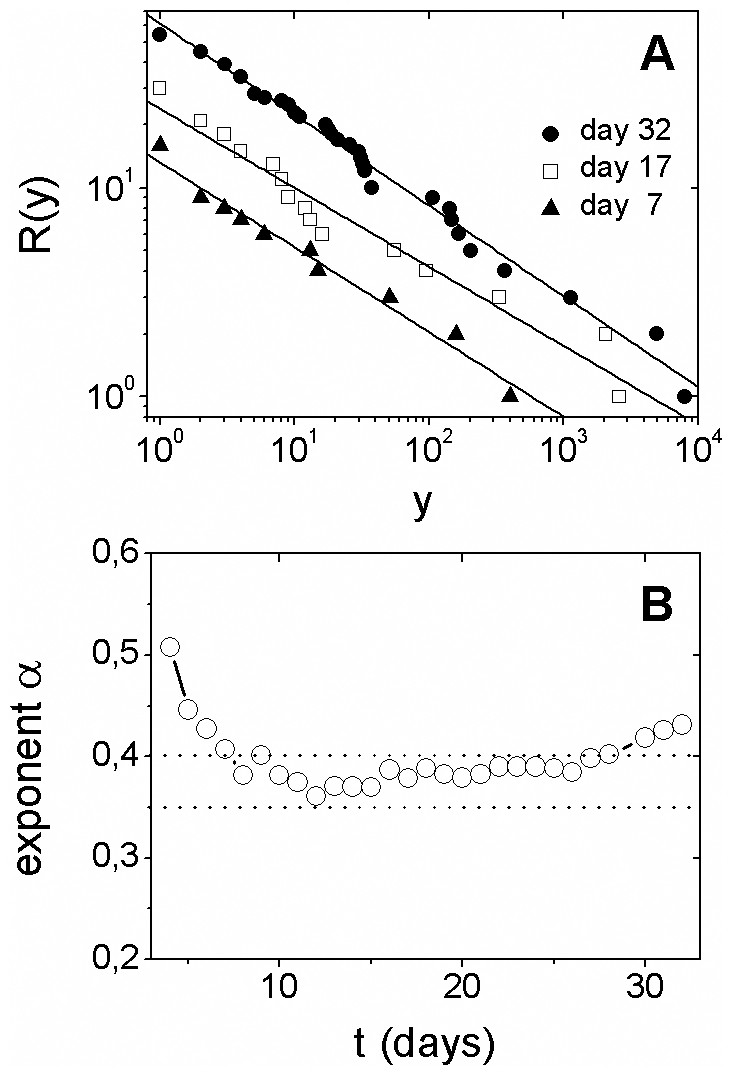
Cumulative distribution of cases among countries: power law behavior. (**A**) Empirical cumulative distribution, *R*(*y*), of laboratory-confirmed cases *y* for distinct times (few specific days are displayed for better visualization). The solid lines are power laws, given by Eq. (1), with exponents following Fig. 2B. (**B**) Temporal evolution of the power law exponent *α*, obtained by least-square fits to the data. Observe that 0.35<*α*<0.40 for a period of about 20 days.

The empirical cumulative distribution *R*(*y*), for initial times, is shown in Fig. 2A. In this range, *R*(*y*) presents linear behavior on log-log scale being well described by a power law decay,

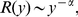
(1)where *α* is the power law exponent. The time evolution of the power law exponent *α* is shown in Fig. 2B. Observe that *α* stays approximately constant, 0.35<*α*<0.040, for 21 days.

**Figure 3:**
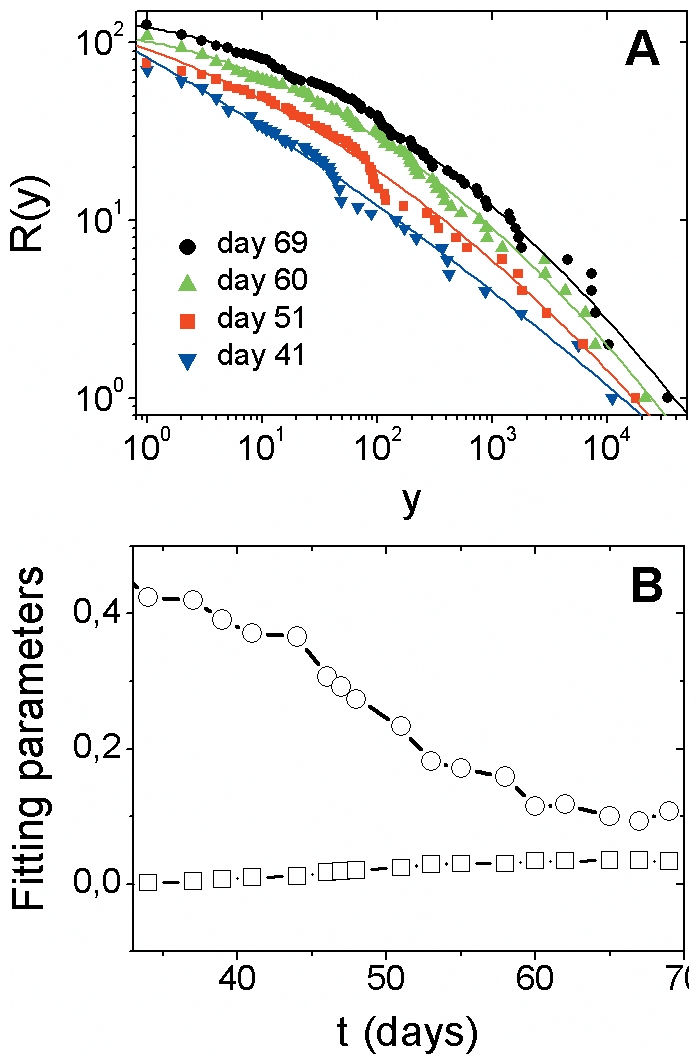
Cumulative distribution of cases among countries: log-normal behavior. (**A**) Empirical cumulative distribution, *R*(*y*), laboratory-confirmed cases *y*, for larger times (for better visualization, we display few specific days). The systematic curvature on log-log scale indicates a gradual deviation from power law behavior. The solid lines are given by Eq. (2), with parameters shown in Fig. 3B. (**B**) Temporal evolution of parameters *α* (circles) and *β* (squares) obtained by least-square fits of Eq. (2) to the data.

For larger times, *R*(*y*) gradually deviates from the power law behavior (Supplementary Figure). In this range, *R*(*y*)has a modest negative curvature everywhere (see Fig. 3A) and is in good agreement with log-normal curves given by
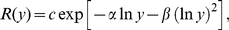
(2)


Where *c* is a normalization constant and *α* and *β* are the parameters. Notice that *R*(*y*) behaves as a power law, 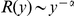, when 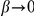. The time evolution of the parameters *α* and *β* of the log-normal curves are shown in Fig. 3B.

## Discussion

The exponential growth shown in Fig. 1A is consistent with typical results in epidemiology. Well known models for the spread of epidemics, such as SEIR model, predicts exponential growth in the number of infections at early stages of the epidemic. A possible explanation of the deviation of exponential behavior for smaller times, also shown in Fig. 1A, may be found in Fig. 1B. The growth of individual countries is added to the growth of the number of countries with new cases. For initial times, when the total number of cases is small, this combination may generate a deviation of the exponential behavior. For larger times, when the number of cases is large, the effect of the entry of new countries with few infections may be much less intense.

According to results shown in Fig. 2, the distribution of cases among countries exhibits power law behavior - indicating scale invariance. The presence of power law behavior suggests that the system self-organizes into a scale-free state. This phenomenon is a remarkable characteristic of a wide range of complex systems - from physics to biology and medicine [12]. Scale invariance in the global spread of influenza pandemic for initial stages is a remarkable feature which is unpredicted by many traditional models for the evolution of infectious diseases. This finding bring to mind a previous study on the distribution of epidemic events in isolated populations [13]. In both cases, the cumulative distribution of events follows a power law behavior, with exponent *α*∼0.3 for isolated populations and 0.035<*α*<0.40 for the 2009 influenza pandemic.

A possible factor contributing to the observed scale invariance is human mobility - which has been considered a fundamental ingredient for infectious diseases to spread rapidly through the world population [14]–[16]. Human mobility may alter the evolution of local epidemics, with the entry of new infectious individuals in a given region, causing a non-local effect [17]. Surprisingly, human travel exhibits scaling laws such as the power law distribution that we find for influenza pandemics [14]. Therefore, it is natural to consider human mobility, including the air traffic in the worldwide air-transportation network, as a possible mechanism contributing for the power law behavior shown in Fig. 2A.

In contrast, other factors may contribute for the deviation from power law behavior observed for larger times. Country-based contingency plans, for example, have been implemented in order to control the international spread of influenza pandemic. Studies have estimated the impact of restricting international travel and imposing entry or exit screening of passengers at airports [18]. Other local contingency plans, which may be a function of the country's level of preparedness to deal with the pandemic, have been implemented in order to reduce the spread of influenza infections [17]. In addition, it is well known that seasonality and weather conditions, such as temperature and relative humidity, affect the dynamics of influenza transmission [19]–[20]. We cannot discard the possibility that such factors, which can largely vary from country to country, may contribute towards changing the shape of the cumulative distribution of cases among countries.

Our findings suggest a crossover in the shape of the cumulative distribution of cases among countries - from power law for initial times to log-normal for larger times. However, we remark that a log-normal distribution can be mistaken locally for a power law. In fact, a log-normal distribution can mimic a power law over a relatively large interval. Concerning its origin, typically log-normal forms underlie random multiplicative process [12] – a pure random multiplicative process is defined as 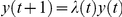, being 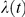 a random number. Thus, factors such as the ones described above may be acting in a multiplicative way in the dynamics of influenza pandemic. This may be a possible origin of the log-normal behavior or *R*(*y*) observed for large times.

Next we present an alternative way to interpret our empirical findings. We compare our results with the predictions of a simple stochastic model. Let us consider that the number of cases in a given country follows a process given by the rule
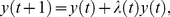
(3)


Where *y*(*t*+1) and *y*(*t*) are the number of cases in the times *t*+1 and *t* respectively, and 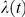 is a random number following an exponential distribution with mean *µ*. Each country evolves independently of the others countries. The system evolves starting from a given initial condition – for example, a particular number of countries with *y*(*t* = 0) =  1. For each time step two new countries are added to the system, each one with probability *p*.

The rule described in Eq. 3 indicates that the number of cases in the time *t*+1 depends of the number of cases in the time *t*. This assumption is compatible with the dynamics of an infectious disease. Each individual infected with influenza virus, for example, has the potential to spread the virus to its neighborhood. Observe also that the random nature of 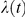 in Eq. (3) is not essential to obtain the results described below. However, the random nature of 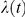 mimics possible fluctuations which are common in real systems.

**Figure 4:**
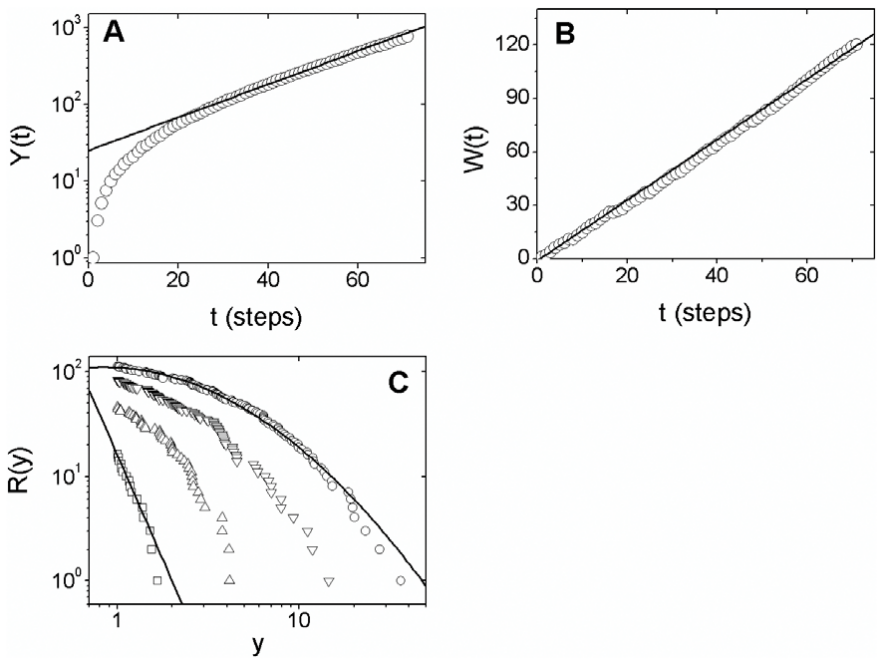
Simulation of the stochastic model. The model is given by Eq. (3), with 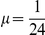 and *p* = 0.85. Initial condition: there are only one country with *y*(*t* = 0) =  1. (**A**) Temporal evolution of the total number of confirmed cases, *Y*(*t*), on mono-log scale, for time steps from 1 to 70. The solid line is given by 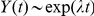, with *λ* = 0.05. (**B**) Temporal evolution of the total number of countries with confirmed cases, *W*(*t*), for the same time steps. The solid line is 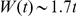. (**C**) Cumulative distribution, *R*(*y*), for *t* = 10 steps (squares), *t* = 30 (up triangles), *t* = 50 (down triangles) and *t* = 70 (circles). For comparison, we show a power law given by Eq. (1), for *t* = 10, and a lognormal curve given by Eq. (2) for *t* = 10 steps.

A typical simulation of this model is shown in Fig. 4 for a particular choice of parameters. Observe that the model predicts qualitatively several aspects of the empirical results – the exponential growth after an initial transient, the linear growth in the number of countries with confirmed cases and a cumulative distribution exhibiting a systematic curvature on log-log scale indicating lognormal behavior.

In this study, we identify spreading patterns of the 2009 influenza A (H1N1/S-OIV) pandemic for a particular time period. Our results provide quantitative evidence of a crossover in the shape of the cumulative distribution of cases – from power law behavior at early stages to log-normal behavior for larger times. We discuss possible factors that may contribute towards changing the shape of the cumulative distribution from power law to log-normal. Country-based contingency plans, country's level of preparedness to deal with the pandemic, seasonal effects, weather conditions among others may be acting in a multiplicative way generating the observed behavior.

Our findings may give us information on the underlying mechanisms governing influenza-type pandemics. For example, the simple stochastic model proposed in this work suggests that the number of confirmed cases in a given country follows a multiplicative process with exponential noise.

The future evolution of any influenza pandemic is difficult to predict. However, the analysis of epidemiological data and the selection of realistic models may provide some insight in the spatial and temporal evolution of pandemic events. Models for the spread of infectious diseases are useful tools - can project plausible scenarios, guide control strategies, suggests roles of antiviral drugs and vaccines and so on. Our empirical results could help to select realistic models of the dynamics of influenza-type pandemic events.

## Supporting Information

Figure S1 **Cumulative distribution of cases among countries for all data.** Empirical cumulative distribution, *R*(*y*), of laboratory-confirmed cases *y* for all data (48 days within the period of 69 days, from 26 April to 3 July, 2009). The data are shown from smaller to larger times – from left to right and from top to bottom. Observe the gradual convergence from a linear behavior on log-log scale (power law behavior) to curves with a modest negative curvature everywhere (log-normal behavior).
(TIF)
