# Supplementary figures and images for: Spreading Patterns of the Influenza A (H1N1) Pandemic
Source: PLoS One. 2011 Mar 31;6(3):e17823. doi: 10.1371/journal.pone.0017823 (PMC3069037; doi:10.1371/journal.pone.0017823)

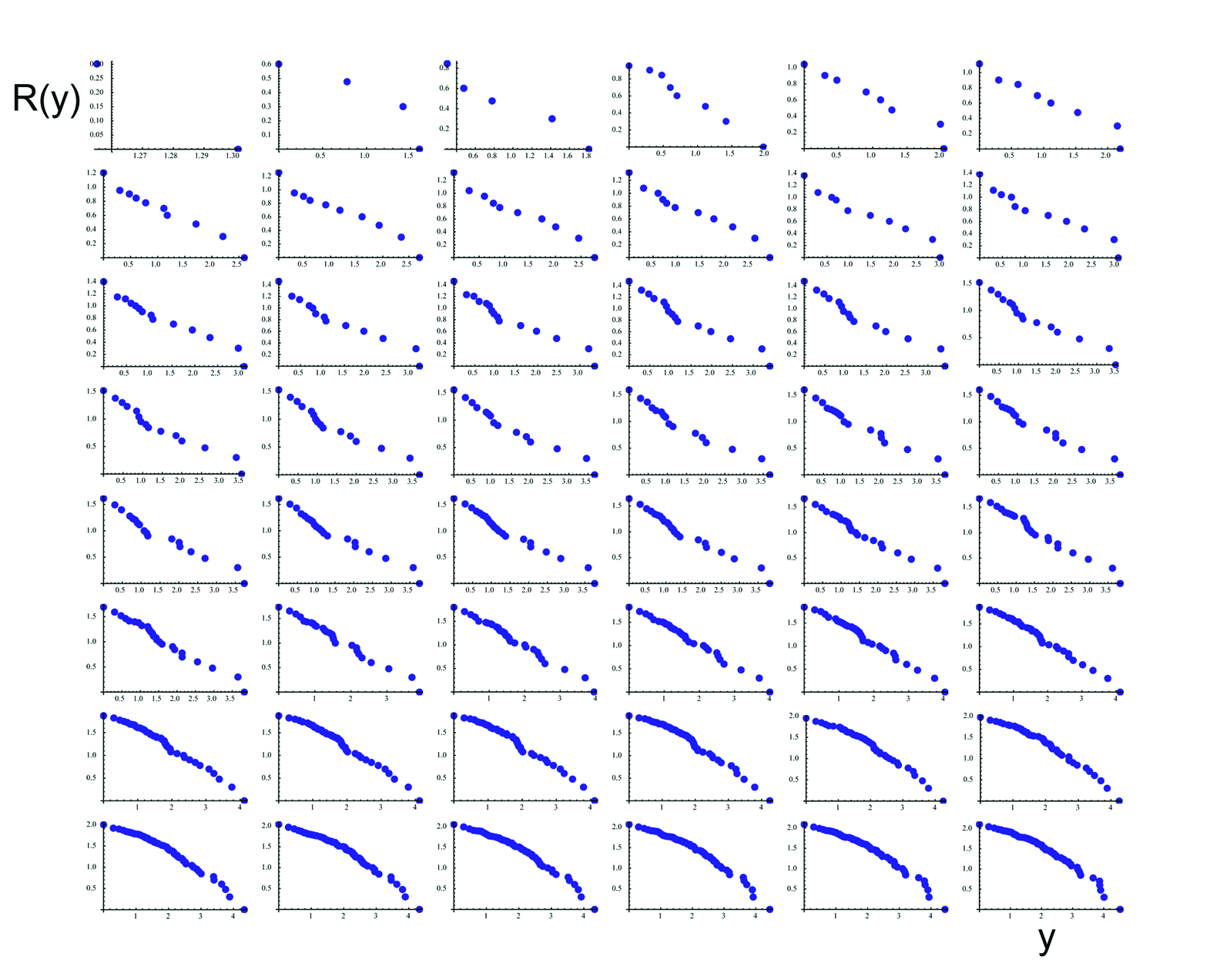

Supplement: Figure S1 — Cumulative distribution of cases among countries for all data. Empirical cumulative distribution, R(y), of laboratory-confirmed cases y for all data (48 days within the period of 69 days, from 26 April to 3 July, 2009). The data are shown from smaller to larger times – from left to right and from top to bottom. Observe the gradual convergence from a linear behavior on log-log scale (power law behavior) to curves with a modest negative curvature everywhere (log-normal behavior). (TIF) [file pone.0017823.s001.tif]
